# Incidence, clinical characteristics, and outcomes of nosocomial *Enterococcus* spp. bloodstream infections in a tertiary-care hospital in Beijing, China: a four-year retrospective study

**DOI:** 10.1186/s13756-017-0231-y

**Published:** 2017-07-04

**Authors:** Yangyang Zhang, Mingmei Du, Yan Chang, Liang-an Chen, Qing Zhang

**Affiliations:** 1 Department of Respiratory Medicine, Chinese PLA General Hospital, Fuxing Road No. 28, Beijing, 100853 China; 2 Department of Respiratory Medicine, Affiliated Hospital of Chengde Medical University, Nanyingzi Street No. 36, Chengde, Hebei Province 067000 China; 3 Department of Infection Management and Disease Control, Chinese PLA General Hospital, Beijing, 100853 China; 4 Department of Respiratory Medicine, The General Hospital of the PLA Rocket Force, Xinjiekou Street No. 16, Beijing, 100088 China

**Keywords:** *Enterococcus*, Nosocomial bloodstream infection, Bacteraemia, Epidemiology, Mortality

## Abstract

**Background:**

*Enterococcus* spp. are the common cause of nosocomial bloodstream infections (BSIs) with high morbidity and mortality. The purpose of this study was to characterize the incidence, clinical and microbiological features, and mortality of nosocomial enterococcal BSIs at a large Chinese tertiary-care hospital in Beijing, China.

**Methods:**

A retrospective cohort study on adult patients with nosocomial BSIs due to *Enterococcus* spp. was performed between January 1, 2012, and December 31, 2015 at the Chinese People’s Liberation Army General Hospital. Patients’ data were gathered by reviewing electronic medical records.

**Results:**

A total of 233 episodes of BSI due to *Enterococcus* spp. occurred among 224 patients during these 4 years. The overall incidence was 3.9 episodes per 10,000 admissions. *Enterococcus faecium* (*E. faecium*) was the major pathogen (74%, 95% CI 68–80%), followed by *Enterococcus faecalis* (*E. faecalis*) (20%, 95% CI 15–25%). *E. faecium* showed higher antimicrobial resistance than *E. faecalis*. The 30-day mortality of nosocomial enterococcal BSI was 24% (95% CI 18–29%). Predictors for mortality included the Acute Physiology and Chronic Health Evaluation II (APACHE II) score, Charlson comorbidity index (CCI), impaired renal function, prior use of immunosuppressive agents, and appropriate empirical antimicrobial treatment.

**Conclusions:**

This study emphasizes that *Enterococcus* spp. were major pathogens for nosocomial BSIs and associated with high mortality. Appropriate empirical antimicrobial treatment can improve outcomes. Vancomycin is the best choice for patients with *E. faecium* BSIs. Penicillins, aminoglycosides, fluoroquinolones, and vancomycin can be considered for patients with *E. faecalis* BSIs.

## Background

BSIs can cause high mortality and result in heavy social and economic burdens. As one of the common gram-positive pathogens, *Enterococcus* spp. are the fourth leading cause of BSIs in North America [1], and are rated as the third most prevalent pathogens of nosocomial BSIs in the United States [2], accounting for >9.0% of the BSIs. Several multicentre studies from China and Japan also reported that *Enterococcus* spp. were the fourth most common BSI pathogens [3–5]. And a systematic review focused on community-acquired BSIs in south and southeast Asia showed that *Enterococcus* spp. were the third most prevalent gram positive bacteria [6]. The crude mortality rates of enterococcal BSIs ranged between 21.4% and 64.2% [7–11]. Risk factors for developing enterococcal BSIs include the presence of comorbidities such as malignancy, diabetes; more severe illness; invasive devices, such as a central intravenous catheter; complicated surgery; solid organ transplantation; and hematopoietic stem cell transplantation [2, 12–16].

Enterococci are innately resistant to cephalosporins, and the commonly used antimicrobial agents such as fluoroquinolones and carbapenems are not recommended. The increasing prevalence of acquired resistance to penicillins and aminoglycosides has been observed in many countries [17, 18]. Vancomycin-resistant enterococci (VRE) are a great challenge in clinical treatment because there are limited bactericidal options to choose [19].

*E. faecalis* and *E. faecium* are the most frequently isolated species, and incidences of both have shown a rising trend, especially for *E. faecium* [11, 20]. *E. faecium* has significantly higher antibiotic resistance rates than *E. faecalis* and may lead to more serious disease and worse outcome [11, 21, 22].

Although there has been much research on enterococcal BSI, the incidence, species distribution, clinical features, and prognosis vary in different periods and different regions. In addition, nosocomial BSI is different from community-acquired BSI in several aspects, including a poor prognosis [11]. Little data exists regarding nosocomial enterococcal BSIs in China. The purpose of the present study was to characterise the incidence, clinical, and microbiological features, and to identify the predictors of crude mortality in patients with enterococcal BSIs at our hospital. The clinical and microbiological characteristics of *E. faecalis* and *E. faecium* were also compared.

## Methods

### Study design, hospital setting, and patients

We performed a retrospective cohort study on adult patients with nosocomial BSIs due to *Enterococcus* spp. between January 1, 2012, and December 31, 2015, at the Chinese People’s Liberation Army General Hospital (PLAGH), a 2200-bed tertiary-level healthcare hospital in Beijing, China. It is one of the biggest comprehensive hospitals in China, with medical, health care, teaching, and scientific research that serves national military and nonmilitary personnel from across the country.

Eligible patients included all patients aged ≥18 years with at least 1 positive blood culture for *Enterococcus* spp. In patients with persistent BSIs caused by the same organism, only the first episode was included. If the patients had 2 or more separate BSIs, each infection was considered individually. All patients were identified by searching the real-time nosocomial infection surveillance system (RT-NISS) [23]. This platform utilises data from electronic medical record systems, such as hospital stay, temperature changes, and microbiology results with the application of clinically validated algorithms to identify and classify all of the patients’ infections. RT-NISS was developed by the Infection Management and Disease Control Department of PLAGH.

### Data collection

The patients’ data were gathered by reviewing electronic medical records. We recorded the demographic data including age and gender. The clinical data collected included: body mass index (BMI), underlying diseases, the CCI score, the APACHE II score in the first 24 h following the onset of BSIs, the hospitalisation wards, previous exposures (prior hospital stay, previous treatment such as surgical procedures, immunosuppressive agents, chemotherapeutic agents, total parenteral nutrition, mechanical ventilation, renal replacement therapy, antibiotics, or invasive devices within 30 days prior to BSIs), treatment (antibiotic choice), and outcomes (length of hospital stay, and all-cause mortality at 7 days and 30 days). The microbiological data collected included: species of *Enterococcus*, likely source of BSIs (identified by treating doctors and/or physicians of the Infection Management and Disease Control Department), whether cultures were polymicrobial, and antimicrobial susceptibility results. We collected the annual admission data to calculate incidence rates, which are expressed as the number of BSI episodes per 10,000 hospital admissions.

### Definitions

The diagnosis of enterococcal BSI should meet the following criteria: 1)isolation of *Enterococcus* spp. from one or more blood cultures; and one of the following 2) fever (>38 °C), chills, or hypotension; and 3) eliminated the possibility of contamination during the collection and cultivation of blood samples [24]. Nosocomial BSI was defined as the first positive blood culture obtained ≥48 h after hospital admission and with no evidence of infection at admission [15, 24]. An episode was defined as the positive isolation of enterococcus from at least one blood culture sample from a patient and without the prior blood culture isolating the same bacteria within the previous 30 days [2, 25]. Onset of BSIs was defined as the date when the blood culture was collected. Polymicrobial BSIs were defined as 2 or more clinically important organisms isolated from 1 single blood culture sample or different blood culture samples within 48 h. Appropriate antimicrobial treatment was defined as an active antimicrobial choice and an adequate dosage within 5 days from the onset. Empirical antimicrobial therapy was defined as the antimicrobial agents given within 24 h after the onset of BSIs. Active agents were confirmed according to the antimicrobial susceptibility test.

### Identification and antibiotic susceptibility testing

Blood was cultured using BacT/ALERT 3D system (Becton-Dickinson, Sparks, MD, USA) in the microbiology laboratory. Species identification was performed using the VITEK 2 system (BioMérieux, Marcy 1′Étoile, France). Antibiotic susceptibility testing was performed using the VITEK 2 system or the Kirby-Bauer Disk Diffusion method (Oxoid, UK) according to the recommendations proposed by the Clinical and Laboratory Standards Institute (CLSI).

### Statistical analysis

Categorical variables were expressed as frequency counts and percentages with 95% confidence interval (95% CI). Continuous variables were expressed as median and interquartile ranges (IQRs). Comparison of categorical variables was performed using Pearson’s chi-squared test or Fisher’s exact test, and comparison of continuous variables was performed using the Mann-Whitney U test. In order to identify the risk factors associated with mortality, a multivariate logistic regression model with backward method was generated to control the effects of confounding variables. Variables statistically related (*p* < 0.10) to mortality in the univariate logistic analyses were chosen to build the multivariate model. Results with a 2-tailed *p*-value < 0.05 were considered to be significant. All statistical analyses were run using SPSS 20.0 software (IBM Corp., Armonk, NY, USA).

## Results

### Incidence and species distribution

In total, 233 episodes of nosocomial BSIs caused by *Enterococcus* spp. occurred among 224 patients during the 4-year study period. Of the 224 patients who developed enterococcal BSIs, 6 had infections with 2 different species >30 days apart and 3 had 2 infections with the same species >30 days apart. Seven patients who had infections with 2 different species of enterococcus isolated within 48 h were excluded because of the interpretative problem.

**Fig. 1:**
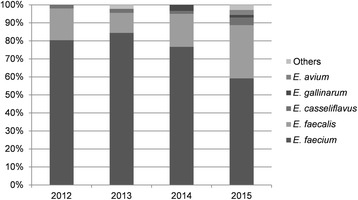
*Enterococcus* species ratio from 2012 to 2015

For the remaining 226 episodes, classification was available in 224 (99%, 95% CI 98–100%). The most common *Enterococcus* species was *E. faecium*, which comprised 167 (74%, 95% CI 68–80%) of all episodes. *E. faecalis* was the second largest species, comprising 46 (20%, 95% CI 15–25%) of episodes. Of the remaining episodes, 6 (3%, 95% CI 1–5%) due to *E. casseliflavus*, 3 (1%, 95% CI 0–3%) due to *E. gallinarum*, and 2 (1%, 95% CI 0–3%) due to *E. avium*. The number of enterococci isolated in 2015 was the highest, and the least was in 2013. The species ratio in each year is shown in Fig. 1.

**Fig. 2:**
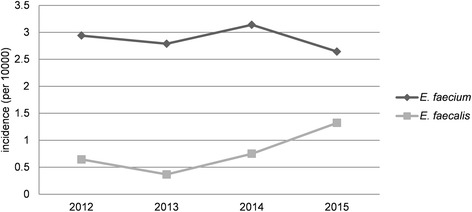
Incidences of BSIs due to *E. faecium* and *E. faecalis* from 2012 to 2015

The overall incidence of nosocomial enterococcal BSIs was 3.9 episodes per 10,000 admissions and the rate fluctuated from 3.3 to 4.4 episodes per 10,000 admissions during the 4 years (3.7 in 2012, 3.3 in 2013, 4.1 in 2014, and 4.4 in 2015). The overall incidence rates of *E. faecium* and *E. faecalis* were 2.9 episodes and 0.8 episodes per 10,000 admissions, respectively. Incidence of *E. faecalis* BSI showed a rising trend, while that of *E. faecium* was on the decline, as shown in Fig. 2.

### Demographic and clinical characteristics

**Fig. 3:**
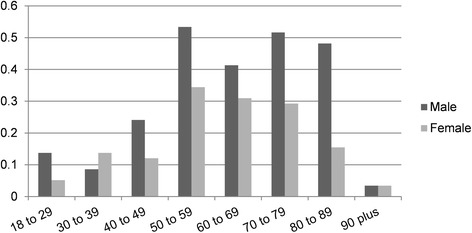
Age and gender distribution of patients with enterococcal BSIs

Demographics and clinical data were available for all these 226 incident episodes as shown in Table 1. The median age was 65 years (IQR, 54–75), and 142 (63%, 95% CI 56–69%) of the patients were male. The age distribution was right skewed, with the peak incidence in the 50–59 years group (Figure 3). Malignancy was the most common comorbidity (58%, 95% CI 51–64%), followed by cardiovascular disease (50%, 95% CI 43–57%) and pneumonia (33%, 95% CI 27–39%). The median and range of BMI were all within the normal range. The median CCI score was 3 (IQR, 2–6), and 86.7% of the patients had combined chronic diseases (CCI score ≥ 1). The median APACHE II score was 8 (IQR, 5–18). Some 172 (76%, 95% CI 71–82%) of the episodes occurred in the non-ICU ward, and 54 (24%, 95% CI 18–29%) occurred in the ICU. The median days of hospital stay prior to and after the onset of BSI were 15 and 18, respectively. A total of 210 (93%, 95% CI 90–96%) episodes had prior antibiotic exposure and 182 (81%, 95% CI 75–86%) had invasive medical procedures within the 30 days prior to BSI, such as a central intravenous catheter, Indwelling urinary catheter, and drainage tube.

**Table 1:** Demographic and clinical characteristics of patients with enterococcal BSIs

	Total (*n* = 226)	*E. faecium* (*n* = 167)	*E. faecalis* (*n* = 46)	*p-*value
Demographics				
Age	65 (54–75)	65 (55–75)	66 (52–79)	0.450
Male gender	142 (63, 56–69)	104 (62, 55–70)	31 (67, 53–81)	0.524
BMI, median	23 (20–26)	22 (20–25)	24 (23–26)	***0.006***
Comorbidities				
Malignancy	130 (58, 51–64)	95 (57, 49–64)	26 (57, 42–71)	0.965
Cardiovascular disease	113 (50, 43–57)	78 (47, 39–54)	28 (61, 46–76)	0.089
Pneumonia	74 (33, 27–39)	60 (36, 29–43)	14 (30, 17–44)	0.488
Diabetes mellitus	68 (30, 24–36)	45 (27, 20–34)	18 (39, 24–54)	0.109
Impaired liver function	52 (23, 17–29)	41 (25, 18–31)	7 (15, 4–26)	0.180
Cerebrovascular disease	41 (18, 13–23)	34 (20, 14–27)	5 (11, 2–20)	0.141
Impaired renal function	30 (13, 9–18)	22 (13, 8–18)	7 (15, 4–26)	0.720
Hemiplegia	27 (12, 8–16)	24 (14, 9–20)	2 (4, 0–10)	0.066
Neutropenia	14 (6, 3–9)	12 (7, 3–11)	2 (4, 0–10)	0.492
CCI, median	3 (2–6)	3 (2–6)	2 (1–4)	***0.014***
APACHE II score, median	8 (5–11)	8 (5–11)	7 (5–12)	0.501
Hospitalization ward				
Medical	88 (39, 33–45)	68 (41, 33–48)	14 (30, 17–44)	0.204
Surgical	84 (37, 31–44)	56 (34, 26–41)	22 (48, 33–63)	0.075
ICU	54 (24, 18–29)	43 (26, 19–32)	10 (22, 9–34)	0.578
Length of hospital stay, median days				
Prior hospital stay	15 (9–27)	16 (10–28)	14 (7–25)	0.065
Hospital stay after onset of BSIs	18 (8–29)	18 (8–29)	18 (8–33)	0.906
Previous treatment				
Antibiotic exposure	210 (93, 90–96)	160 (96, 93–99)	41 (89, 80–98)	0.082
Total parenteral nutrition	84 (37, 31–44)	60 (36, 29–43)	22 (48, 33–63)	0.142
Mechanical ventilation	87 (38, 32–45)	63 (38, 30–45)	20 (43, 29–58)	0.479
Surgery	74 (33, 27–39)	49 (29, 22–36)	20 (43, 29–58)	0.070
Chemotherapeutic agent	29 (13, 8–17)	24 (14, 9–20)	3 (7, 0–14)	0.153
Immunosuppressive agent	18 (8, 4–12)	16 (10, 5–14)	2 (4, 2–10)	0.259
Renal replacement therapy	15 (7, 3–10)	12 (7, 3–11)	3 (7, 0–14)	0.876
Invasive devices				
Central intravenous catheter	102 (45, 39–52)	82 (49, 41–57)	16 (35, 20–49)	0.084
Indwelling urinary catheter	104 (46, 39–53)	75 (45, 37–53)	25 (54, 39–69)	0.256
Endotracheal intubation	86 (38, 32–44)	60 (36, 29–43)	22 (48, 33–63)	0.142
Peripheral intravenous catheter	69 (31, 24–37)	50 (30, 23–37)	14 (30, 17–44)	0.948
Tracheostomy tube	20 (9, 5–13)	15 (9, 5–13)	4 (9, 0–17)	0.952

Data are presented as n (%,95% CI) or median (IQR)

Significant variables are appeared in bold and italics text

A comparison of nosocomial BSIs according to *E. faecium* and *E. faecalis* in demographics and clinical characteristics is shown in Table 1. Patients with *E. faecalis* BSIs presented a higher BMI, but the median was in the normal range. Patients with *E. faecium* BSIs showed a higher CCI score than those with *E. faecalis* BSIs (median, 3 vs 2, *p* = 0.014). The median APACHE II score of patients with *E. faecium* BSIs was higher than in those with *E. faecalis* BSIs, but these were not statistically significant.

### Microbiology and antimicrobial therapy

The source of nosocomial enterococcal BSIs was mainly related to intra-abdominal and central venous catheter, with the number of 92 (41%, 95% CI 34–47%) and 82 (36%, 95% CI 30–43%), respectively. Foci could not be confirmed for 65 (29%, 95% CI 23–35%) episodes. A total of 21 (9%, 95% CI 5–13%) episodes had polymicrobial infections.

The microbiological data of BSIs due to *E. faecium* and *E. faecalis* are presented in Table 2. There was no difference in the sources of BSI between these 2 species. Patients with *E. faecium* BSIs were more likely to have polymicrobial infections than those with *E. faecalis* (12% vs 2%, *p* = 0.048). No vancomycin-resistant *E. faecalis* isolate was found, and 7 (4%, 95% CI 1–7%) vancomycin- resistant *E. faecium* isolates were identified. *E. faecium* isolates had higher resistance rates than *E. faecalis* to ampicillin (86% vs 9%, *p* < 0.001), erythromycin (78% vs 56%, *p* = 0.009), and ciprofloxacin (86% vs 39%, *p* < 0.001).

**Table 2:** Comparison of the microbiological characteristics and treatment of patients with *E. faecium* and *E. faecalis* BSIs

	*E. faecium* (*n* = 167)	*E. faecalis* (*n* = 46)	*p*-value
Source of BSIs			
Intra-abdominal	65 (39, 31–46)	19 (41, 27–56)	0.770
Unknown	53 (32, 25–39)	10 (22, 9–34)	0.188
Central venous catheter	30 (18, 12–24)	10 (22, 9–34)	0.562
Genitourinary	11 (7, 3–10)	3 (7, 0–14)	0.987
Pneumonia	4 (2, 0–5)	3 (7, 0–14)	0.165
Others	4 (2, 0–5)	1 (2, 0–7)	0.930
Type of BSIs			
Polymicrobial	20 (12, 7–17)	1 (2, 0–7)	***0.048***
Antibiotic resistance^a^			
Ampicillin (137 vs 46)^b^	118 (86, 80–92)	4 (9, 0–7)	***<0.001***
Gentamicin (93 vs 21) ^b^	49 (53, 42–63)	7 (33, 11–55)	0.109
Tetracycline (105 vs 36) ^b^	50 (48, 38–57)	22 (61, 44–78)	0.162
Erythromycin (105 vs 36) ^b^	82 (78, 70–86)	20 (56, 39–73)	***0.009***
Ciprofloxacin (137 vs 46) ^b^	118 (86, 80–92)	18 (39, 24–54)	***<0.001***
Vancomycin (167 vs 46) ^b^	7 (4, 1–7)	0 (0, 0)	0.158
Treatment after the onset of BSIs			
Appropriate antimicrobial treatment	157 (94, 90–98)	43 (93, 86–100)	0.893
Appropriate empirical treatment	62 (37, 30–45)	26 (57, 42–71)	***0.018***

Data are presented as n (%,95% CI) or median (IQR)

Significant variables are appeared in bold and italics text

^a^Not all agents listed tested in all isolates

^b^The numbers in parentheses represent the total numbers of *E. faecium* and *E. faecalis* isolates performed susceptibility test

Before obtaining the report of antibiotic susceptibility test reports, 98 (43%, 95% CI 37–50%) patients were treated with effective antibiotics. Patients with *E. faecalis* BSIs were more likely to get effective treatment by the empirical use of antibiotics (57% vs 37%, *p* = 0.018).

### Outcomes

The median length of hospital stay was 34 days (IQR, 23–55). There was no statistical difference in the length of stay between patients with *E. faecium* BSIs (median, 35) and those with *E. faecalis* BSIs (median, 34). The total 7-day and 30-day mortality rates were 11% (95% CI 7–15%) and 24% (95% CI 18–29%), respectively. The 7-day and 30-day mortality rates for *E. faecium* BSIs were higher than *E. faecalis* BSIs (13% vs 9%, 25% vs 17%), but both had no statistical significance. Patients with malnutrition showed a higher risk of 7-day and 30-day mortality than patients with normal BMI.

Univariate analyses of predictors for 7-day mortality and 30-day mortality are shown separately in Table 3. The 7-day mortality was associated with pneumonia, impaired renal function, CCI, APACHE II score, ICU residence, prior exposure to immunosuppressive agents and central intravenous catheter, and appropriate empirical antimicrobial treatment. The predictors for 30-day mortality were similar to those for 7-day mortality, except for surgery and renal replacement therapy prior to the onset of BSI within 30 days.

**Table 3:** Univariate logistic regression analysis of risk factors for mortality

Variable	7-day mortality				30-day mortality			
	Survivors (*n* = 201)	Non-survivors (*n* = 25)	OR (95% CI)	*p*-value	Survivors (*n* = 172)	Non-survivors (*n* = 54)	OR (95% CI)	*p*-value
Age, ≥ 70 years	77 (38, 32–45)	13 (52, 31–71)	1.7 (0.8–4.0)	0.191	65 (38, 30–45)	25 (46, 33–60)	1.4 (0.8–2.6)	0.266
Male gender	130 (65, 58–71)	12 (48, 27–69)	0.5 (0.2–1.2)	0.108	113 (66, 59–73)	29 (54, 40–67)	0.6 (0.3–1.1)	0.113
BMI				0.181				0.118
Normal	121 (60, 53–67)	11 (44, 23–65)	1.0		104 (60, 53–68)	28 (52, 38–66)	1.0	
Malnutrition	19 (9, 5–14)	6 (24, 6–42)	3.5 (1.2–10.5)	0.027	14 (8, 4–12)	11 (20, 9–31)	2.9 (1.2–7.1)	0.019
Overweight	53 (26, 20–33)	7 (28, 9–47)	1.5 (0.5–4.0)	0.465	47 (27, 21–34)	13 (24, 12–36)	1.0 (0.5–2.2)	0.943
Obese	8 (4, 1–7)	1 (4, 0–12)	1.4 (0.2–12.0)	0.773	7 (4, 1–7)	2 (4, 0–9)	1.1 (0.2–5.4)	0.943
Comorbidities								
Malignancy	118 (59, 52–66)	12 (48, 27–69)	0.7 (0.3–1.5)	0.310	99 (58, 50–65)	31 (57, 44–71)	1.0 (0.5–1.8)	0.984
Cardiovascular disease	99 (49, 42–56)	14 (56, 35–77)	1.3 (0.6–3.0)	0.526	85 (49, 42–57)	28 (52, 38–66)	1.1 (0.6–2.0)	0.755
Pneumonia	58 (29, 23–35)	16 (64, 44–84)	4.4 (1.8–10.5)	***0.001*** ^a^	47 (27, 21–34)	27 (50, 36–64)	2.7 (1.4–5.0)	***0.002*** ^b^
Diabetes mellitus	58 (29, 23–35)	10 (40, 19–61)	1.6 (0.7–3.9)	0.255	46 (27, 20–33)	22 (41, 27–54)	1.9 (1.0–3.6)	0.052^b^
Impaired liver function	43 (21, 16–27)	9 (36, 16–56)	2.1 (0.9–5.0)	0.107	36 (21, 15–27)	16 (30, 17–42)	1.6 (0.8–3.2)	0.187
Cerebrovascular disease	37 (18, 13–24)	4 (16, 1–31)	0.8 (0.3–2.6)	0.768	31 (18, 12–24)	10 (19, 8–29)	1.0 (0.5–2.3)	0.934
Impaired renal function	22 (11, 7–15)	8 (32, 12–52)	3.8 (1.5–9.9)	***0.006*** ^a^	14 (8, 4–12)	16 (30, 17–42)	4.8 (2.1–10.6)	***0.000*** ^b^
Hemiplegia	25 (12, 8–17)	2 (8, 0–19)	0.6 (0.1–2.8)	0.523	22 (13, 8–18)	5 (9, 1–17)	0.7 (0.3–1.9)	0.487
Neutropenia	12 (6, 3–9)	2 (8, 0–19)	1.4 (0.3–6.5)	0.692	8 (5, 1–8)	6 (11, 2–20)	2.6 (0.9–7.8)	0.095^b^
CCI	3 (2–5)	4 (3–6)	1.2 (1.0–1.4)	***0.028*** ^a^	3 (2–5)	4 (3–6.25)	1.3 (1.1–1.4)	***<0.001*** ^b^
APACHE II score	7 (5–10)	14 (12–17)	1.2 (1.1–1.3)	***<0.001*** ^a^	6 (5–8.75)	13 (10–17)	1.3 (1.2–1.4)	***<0.001*** ^b^
ICU residence	53 (26, 20–33)	13 (52, 31–73)	3.0 (1.3–7.0)	***0.010*** ^a^	42 (24, 18–31)	24 (44, 31–58)	2.5 (1.3–4.7)	***0.005*** ^b^
Previous treatment								
Antibiotic exposure	185 (92, 88–96)	25 (100)		0.998	156 (91, 86–95)	54 (100)		0.998
Total parenteral nutrition	74 (37, 30–44)	10 (40, 19–61)	1.1 (0.5–2.7)	0.756	58 (34, 27–41)	26 (48, 34–62)	1.8 (1.0–3.4)	0.057^b^
Mechanical ventilation	77 (38, 32–45)	10 (40, 19–61)	1.1 (0.5–2.5)	0.870	66 (38, 31–46)	21 (39, 25–52)	1.0 (0.6–1.9)	0.946
Surgery	69 (34, 28–41)	5 (20, 3–37)	0.5 (0.2–1.3)	0.157	63 (37, 29–44)	11 (20, 9–31)	0.4 (0.2–0.9)	***0.029*** ^b^
Chemotherapeutic agent	27 (13, 9–18)	2 (8, 0–19)	0.6 (0.1–2.5)	0.449	24 (14, 9–19)	5 (9, 1–17)	0.6 (0.2–1.7)	0.372
Immunosuppressive agent	13 (6, 3–10)	5 (20, 3–37)	3.6 (1.2–11.2)	***0.026*** ^a^	9 (5, 2–9)	9 (17, 6–27)	3.6 (1.4–9.7)	***0.010*** ^b^
Renal replacement therapy	11 (5, 2–9)	4 (16, 1–31)	3.3 (1.0–11.3)	0.058^a^	8 (5, 1–8)	7 (13, 4–22)	3.1 (1.1–8.9)	***0.040*** ^b^
Invasive devices								
Central intravenous catheter	86 (43, 36–50)	16 (64, 44–84)	2.4 (1.0–5.6)	***0.049*** ^a^	71 (41, 34–49)	31 (57, 44–71)	1.9 (1.0–3.6)	***0.039*** ^b^
Dwell time of central intravenous catheter	15 (7, 26)	14 (7, 24)	1.0 (1.0–1.0)	0.919	15 (7.5, 26.75)	14 (6.5, 23.25)	1.0 (1.0–1.0)	0.465
Indwelling urinary catheter	90 (45, 38–52)	14 (56, 35–77)	1.6 (0.7–3.6)	0.291	76 (44, 37–52)	28 (52, 38–66)	1.4 (0.7–2.5)	0.325
Endotracheal intubation	76 (38, 31–45)	10 (40, 19–61)	1.1 (0.5–2.6)	0.832	65 (38, 30–45)	21 (39, 25–52)	1.1 (0.6–2.0)	0.885
Peripheral intravenous catheter	62 (31, 24–37)	7 (28, 9–47)	0.9 (0.4–2.2)	0.771	53 (31, 24–38)	16 (30, 17–42)	1.0 (0.5–1.8)	0.869
Tracheostomy tube	19 (9, 5–14)	1 (4, 0–12)	0.4 (0.1–3.1)	0.381	16 (9, 5–14)	4 (7, 0–15)	0.8 (0.3–2.4)	0.670
Enterococcal species				0.772				0.175
*E. faecium*	146 (73, 66–79)	21 (84, 69–99)	1.0		122 (71, 64–78)	45 (83, 73–94)	1.0	
*E. faecalis*	42 (20, 15–26)	4 (16, 1–31)	0.7 (0.2–2.0)	0.472	38 (22, 16–28)	8 (15, 5–25)	0.6 (0.3–1.3)	0.188
Others	13 (6, 3–10)	0 (0)	0.0	0.999	12 (7, 3–11)	1 (2, 0–6)	0.2 (0.0–1.8)	0.159
Source of BSIs								
Abdominal	83 (41, 34–48)	9 (36, 16–56)	0.8 (0.3–1.9)	0.612	72 (42, 34–49)	20 (37, 24–50)	0.8 (0.4–1.5)	0.529
Unknown	56 (28, 22–34)	9 (36, 16–56)	1.5 (0.6–3.5)	0.399	47 (27, 20–33)	18 (33, 20–46)	1.3 (0.7–2.6)	0.396
Central venous catheter	37 (18, 13–24)	4 (16, 1–31)	0.8 (0.3–2.6)	0.768	33 (19, 13–25)	8 (15, 5–25)	0.7 (0.3–1.7)	0.468
Genitourinary	12 (6, 3–9)	2 (8, 0–19)	1.4 (0.3–6.5)	0.692	9 (5, 2–9)	5 (9, 1–17)	1.9 (0.6–5.8)	0.281
Pneumonia	7 (3, 1–6)	1 (4, 0–12)	1.2 (0.1–9.8)	0.895	7 (4, 1–7)	1 (2, 2–6)	0.5 (0.1–3.7)	0.453
Polymicrobial infection	18 (9, 5–13)	3 (12, 0–26)	1.4 (0.4–5.1)	0.622	14 (8, 4–12)	7 (13, 4–22)	1.7 (0.6–4.4)	0.291
Vancomycin-resistant	7 (3, 1–6)	0 (0)	0.0	0.999	5 (3, 0–5)	2 (4, 0–9)	1.3 (0.2–6.8)	0.769
Appropriate emperical antimicrobial treatment	200 (100, 99–100)	13 (52, 31–73)	0.0 (0.0–0.1)	***<0.001*** ^a^	172 (100)	41 (76, 64–88)	0.2 (0.1–0.5)	***<0.001*** ^b^

Data are presented as n (%,95% CI) or median (IQR)

Significant variables are appeared in bold and italics text

^a^Variables entered into multivariable logistic regression model of risks factors for 7-day mortality

^b^Variables entered into multivariable logistic regression model of risks factors for 30-day mortality

In the multivariate logistic regression model (Table 4), the risk factors for 7-day mortality included increasing APACHE II scores (OR 1.2, 95% CI 1.1–1.3), and the use of immunosuppressive agents prior to the onset of enterococcal BSIs (OR 4.6, 95% CI 1.1–19.2); the risk factors for 30-day mortality included impaired renal function (OR 3.3, 95% CI 1.1–9.8), high CCI (OR 1.3, 95% CI 1.1–1.6) and APACHE II scores (OR 1.3, 95% CI 1.2–1.4), and prior exposure to immunosuppressive agents (OR 7.3, 95% CI 1.8–29.0). Appropriate empirical antimicrobial therapy was a protective factor for both 7-day (OR 0.2, 95% CI 0.1–0.7) and 30-day mortality (OR 0.2, 95% CI 0.1–0.4).

**Table 4:** Multivariate logistic regression models of risk factors for mortality

Variable	7-day mortality		30-day mortality	
	OR (95% CI)	*p*-value	OR (95% CI)	*p*-value
Diabetes mellitus	-	-	2.1 (0.9–5.4)	0.103
Impaired renal function	2.1 (0.6–6.9)	0.220	3.3 (1.1–9.8)	***0.030*** ^b^
CCI	1.2 (0.9–1.5)	0.080	1.3 (1.1–1.6)	***0.003*** ^b^
APACHE II score	1.2 (1.1–1.3)	***<0.001*** ^a^	1.3 (1.2–1.4)	***<0.001*** ^b^
Prior use of immunosuppressive agents	4.6 (1.1–19.2)	***0.036*** ^a^	7.3 (1.8–29.0)	***0.005*** ^b^
Appropriate empirical antimicrobial therapy	0.2 (0.0–0.7)	***0.010*** ^a^	0.2 (0.1–0.4)	***<0.001*** ^b^

Significant variables are appeared in bold and italics text

^a^Variables entered the final multivariable logistic regression model of risks factors for 7-day mortality

^b^Variables entered the final multivariable logistic regression model of risks factors for 30-day mortality

## Discussion

This study focused on the incidence and characteristics of nosocomial enterococcal BSI in one of the biggest comprehensive hospitals in China. Two multicentre studies in China reporting on the species distribution and antibiotic resistance of clinical isolates from blood cultures showed that *Enterococcus* spp. were the fourth most common pathogens [3, 4]. But, these 2 studies did not involve the species distribution, clinical information, and prognosis. A single-centre study published recently, also devoted to explore the features of *Enterococcus* spp. BSI in a teaching hospital of China [26]. But the sample size (64 episodes) was smaller than our study, and all nosocomial, health care associated, and community acquired enterococcal BSIs were included.

In our 4-year study, the incidence rate of nosocomial enterococcal BSIs fluctuated from 3.3 to 4.4 episodes per 10,000 admissions. This is similar to an earlier study conducted from 1995 to 2002 at 42 US hospitals [2], but is higher than 2 recent reports from Denmark (1.96/10,000) and Canada (0.69/10,000) [11, 15]. But these were both population-based studies. To our knowledge, there are no such reference data about the incidence of nosocomial enterococcal BSIs in China.

In accordance with other studies, patients with enterococcal BSIs were commonly associated with complications, such as malignant, cardiovascular diseases, diabetes mellitus, or chronic kidney disease [11, 15, 22, 27]. We found that pneumonia was also a major comorbidity of nosocomial enterococcal BSIs at our hospital.

*E. faecalis* was reported to be the most common pathogen for nosocomial enterococcal BSIs in most investigations, and the ratio of *E. faecium* was no more than 50% [1, 11, 20, 28]. However, a constant increase in *E. faecium* BSI rates was observed [11, 29], and the incidence of *E. faecium* BSIs exceeded *E. faecalis* BSI in 2009 in a 14-year study conducted at a Swiss tertiary hospital [29]. At our hospital, the incidence of nosocomial *E. faecium* BSIs was significantly higher than that of *E. faecalis* BSIs (2.9/10,000 vs 0.8/10,000). The total vancomycin resistance rate of all isolates was 3%, and 4% for *E. faecium* in our study. All of the vancomycin-resistant isolates were *E. faecium*. This vancomycin resistance rate is higher than those in the Danish study (1.9%) [11], but lower than many studies in other countries. The SCOPE project carried out between 1995 and 1996 at 49 US hospitals reported the VRE rate was between 9.5% to 20.6% in nosocomial enterococcal BSIs from different hospitals [30]. The 1997 SENTRY program reported that 14.1% of enterococcal BSIs were VRE in the United States [1]. A prospective nationwide surveillance study at Brazilian hospitals, which published in 2011, showed that the VRE rate was as high as 25% [9]. Therefore, the species distribution and antimicrobial resistance varies geographically.

Abdominal infections, central venous catheters, and unknown sources were the most common foci of enterococcal BSIs in the present study, is similar to the prior studies [28]. However, no infective endocarditis was observed in our study. There may be 2 reasons to explain this difference. First, the study population was different, as we were focused on nosocomial infection. Another reason was that echocardiography was not a routine examination for patients with BSIs in our hospital, so this could lead to missed diagnosis.

Our data showed that patients with nosocomial *E. faecalis* BSIs were more likely to get appropriate empirical treatment than those with *E. faecium* BSIs. This may be explained by the low resistance rate of *E. faecalis* to many antimicrobial agents, such as ampicillin (9%), gentamicin (33%), and ciprofloxacin (39%).

In the present study, the 30-day mortality of nosocomial enterococcal BSIs was 24%, and the early mortality (7-day) accounted for half. This is lower than many prior reports [5, 8, 9, 12, 31], but similar to a study focusing on vancomycin-susceptible enterococcal BSIs [22]. This may be due to selection bias of the study population, or may be a reflection of medical progress. Mortality was higher in patients with higher CCI and APACHE II scores, prior use of immunosuppressive agents, and complicated with impaired renal function. In other reports, the risk factors for mortality of patients with enterococcal BSIs may also include advanced age, pulmonary infection, malignancy, species, polymicrobial infection, ampicillin resistance, high-level gentamicin resistance, and vancomycin resistance [11, 15, 22]. Although controversial, we confirm that the severity of underlying diseases is the most important factor, especially for early morbidity.

The present study has several limitations that should be taken into consideration. First, our study was a retrospective study, the collection of clinical data depended on medical records rather than interviews and clinical examinations at the onset of infection by unified training doctors of our research group. Second, as a single-centre study, it could lead to an inevitable selection bias, and the multivariate logistic analysis might be affected by the small sample size. Third, not all isolates did all the same antimicrobial agent sensitivity test, so we could not incorporate all the antimicrobial agents resistance in predicting mortality risk factors in order to avoid selection bias.

## Conclusions

Enterococci were major pathogens for nosocomial BSIs and were associated with high mortality,especially for patients combined with chronic diseases. Increased CCI and APACHE II scores, prior use of immunosuppressive agent and complicated with impaired renal function were risk factors for morality. *E. faecium* were more common than *E. faecalis* at our hospital. For patients with *E. faecium* BSIs who conditions were complicated with serious underlying diseases, vancomycin is the best choice; for patients with *E. faecalis* BSIs, penicillins, aminoglycosides, and fluoroquinolones could also be considered besides vancomycin.

## Abbreviations

APACHE II: Acute Physiology and Chronic Health Evaluation II
BMI: Body mass index
BSIs: Bloodstream infections
CCI: Charlson comorbidity index
CI: Confidence interval
IQRs: Interquartile ranges
OR: Odds ratio
PLAGH: Chinese People’s Liberation Army General Hospital
RT-NISS: Real-time nosocomial infection surveillance system
VRE: Vancomycin-resistant enterococci

## References

[1] Pfaller MA, Jones RN, Doern GV, Sader HS, Kugler KC, Beach ML (1999). Survey of blood stream infections attributable to gram-positive cocci: frequency of occurrence and antimicrobial susceptibility of isolates collected in 1997 in the United States, Canada, and Latin America from the SENTRY antimicrobial surveillance program. SENTRY participants group. Diagn Microbiol Infect Dis.

[2] Wisplinghoff H, Bischoff T, Tallent SM, Seifert H, Wenzel RP, Edmond MB (2004). Nosocomial bloodstream infections in US hospitals: analysis of 24,179 cases from a prospective nationwide surveillance study. Clin Infect Dis.

[3] Huang YC, Xie Y, Chen ZX, Liu B, Fei Y, Du Y, Shan B, Kang M (2015). Bloodstream infections in southwestern China: 2012 Whire union report on bacterial susceptibility to antibiotics. Sichuan da xue xue bao Yi xue ban.

[4] Guanghui ZD LI, Fu WANG, Zhidong HU, Quan LI, Ziyong SUN (2014). The distribution and antibiotic resistance of clinical isolates from blood culture in 2012 CHINET surveillance program in China. Chin J Chemother.

[5] Nagao M (2013). A multicentre analysis of epidemiology of the nosocomial bloodstream infections in Japanese university hospitals. Clin Microbiol Infect.

[6] Deen J, von Seidlein L, Andersen F, Elle N, White NJ, Lubell Y (2012). Community-acquired bacterial bloodstream infections in developing countries in south and southeast Asia: a systematic review. Lancet Infect Dis.

[7] DiazGranados CA, Zimmer SM, Klein M, Jernigan JA (2005). Comparison of mortality associated with vancomycin-resistant and vancomycin-susceptible enterococcal bloodstream infections: a meta-analysis. Clin Infect Dis.

[8] Caballero-Granado FJ, Becerril B, Cuberos L, Bernabeu M, Cisneros JM, Pachon J (2001). Attributable mortality rate and duration of hospital stay associated with enterococcal bacteremia. Clin Infect Dis.

[9] Marra AR, Camargo LF, Pignatari AC, Sukiennik T, Behar PR, Medeiros EA, Ribeiro J, Girao E, Correa L, Guerra C, Brites C, Pereira CA, Carneiro I, Reis M, de Souza MA, Tranchesi R, Barata CU, Edmond MB (2011). Nosocomial bloodstream infections in Brazilian hospitals: analysis of 2,563 cases from a prospective nationwide surveillance study. J Clin Microbiol.

[10] Linden PK, Pasculle AW, Manez R, Kramer DJ, Fung JJ, Pinna AD, Kusne S (1996). Differences in outcomes for patients with bacteremia due to vancomycin-resistant Enterococcus faecium or vancomycin-susceptible E. Faecium. Clin Infect Dis.

[11] Pinholt M, Ostergaard C, Arpi M, Bruun NE, Schonheyder HC, Gradel KO, Sogaard M, Knudsen JD (2014). Incidence, clinical characteristics and 30-day mortality of enterococcal bacteraemia in Denmark 2006-2009: a population-based cohort study. Clin Microbiol Infect.

[12] Noskin GA, Peterson LR, Warren JR (1995) Enterococcus faecium and Enterococcus faecalis bacteremia: acquisition and outcome. Clin Infect Dis 1995;20(2):296-301. 10.1093/clinids/20.2.296 7742433

[13] Peel T, Cheng AC, Spelman T, Huysmans M, Spelman D (2012). Differing risk factors for vancomycin-resistant and vancomycin-sensitive enterococcal bacteraemia. Clin Microbiol Infect.

[14] Tavadze M, Rybicki L, Mossad S, Avery R, Yurch M, Pohlman B, Duong H, Dean R, Hill B, Andresen S, Hanna R, Majhail N, Copelan E, Bolwell B, Kalaycio M, Sobecks R (2014). Risk factors for vancomycin-resistant enterococcus bacteremia and its influence on survival after allogeneic hematopoietic cell transplantation. Bone Marrow Transplant.

[15] Billington EO, Phang SH, Gregson DB, Pitout JD, Ross T, Church DL, Laupland KB, Parkins MD (2014). Incidence, risk factors, and outcomes for Enterococcus spp. blood stream infections: a population-based study. Int J Infect Dis.

[16] Berenger BM, Doucette K, Smith SW (2016). Epidemiology and risk factors for nosocomial bloodstream infections in solid organ transplants over a 10-year period. Transpl Infect Dis.

[17] Bush LM, Calmon J, Cherney CL, Wendeler M, Pitsakis P, Poupard J, Levison ME, Johnson CC (1989). High-level penicillin resistance among isolates of enterococci. Implications for treatment of enterococcal infections. Ann Intern Med.

[18] Patterson JE, Zervos MJ (1990). High-level gentamicin resistance in Enterococcus: microbiology, genetic basis, and epidemiology. Rev Infect Dis.

[19] Barber KE, King ST, Stover KR, Pogue JM (2015). Therapeutic options for vancomycin-resistant enterococcal bacteremia. Expert Rev Anti-Infect Ther.

[20] Bhavnani SM, Drake JA, Forrest A, Deinhart JA, Jones RN, Biedenbach DJ, Ballow CH (2000). A nationwide, multicenter, case-control study comparing risk factors, treatment, and outcome for vancomycin-resistant and -susceptible enterococcal bacteremia. Diagn Microbiol Infect Dis.

[21] Conde-Estevez D, Grau S, Albanell J, Terradas R, Salvado M, Knobel H (2011). Clinical characteristics and outcomes of patients with vancomycin-susceptible Enterococcus faecalis and Enterococcus faecium bacteraemia in cancer patients. Eur J Clin Microbiol Infect Dis.

[22] McBride SJ, Upton A, Roberts SA (2010). Clinical characteristics and outcomes of patients with vancomycin-susceptible Enterococcus faecalis and Enterococcus faecium bacteraemia--a five-year retrospective review. Eur J Clin Microbiol Infect Dis.

[23] Du M, Xing Y, Suo J, Liu B, Jia N, Huo R, Chen C, Liu Y (2014). Real-time automatic hospital-wide surveillance of nosocomial infections and outbreaks in a large Chinese tertiary hospital. BMC Med Inform Decis Mak.

[24] Garner JS, Jarvis WR, Emori TG, Horan TC, Hughes JM (1988). CDC definitions for nosocomial infections. Am J Infect Control.

[25] Satlin MJ, Soave R, Racanelli AC, Shore TB, van Besien K, Jenkins SG, Walsh TJ (2014). The emergence of vancomycin-resistant enterococcal bacteremia in hematopoietic stem cell transplant recipients. Leuk Lymphoma.

[26] Zheng JX, Li H, Pu ZY, Wang HY, Deng XB, Liu XJ, Deng QW, Yu ZJ (2017). Bloodstream infections caused by Enterococcus spp: A10-year retrospective analysis at a tertiary hospital in China. J Huazhong Univ Sci Technolog Med Sci.

[27] Vigani AG, Oliveira AM, Bratfich OJ, Stucchi RS, Moretti ML (2008). Clinical, epidemiological, and microbiological characteristics of bacteremia caused by high-level gentamicin-resistant Enterococcus faecalis. Braz J Med Biol Res.

[28] Hamada Y, Magarifuchi H, Oho M, Kusaba K, Nagasawa Z, Fukuoka M, Yamakuchi H, Urakami T, Aoki Y (2015). Clinical features of enterococcal bacteremia due to ampicillin-susceptible and ampicillin-resistant enterococci: an eight-year retrospective comparison study. J Infect Chemother.

[29] Weisser M, Capaul S, Dangel M, Elzi L, Kuenzli E, Frei R, Widmer A (2013). Additive effect of Enterococcus faecium on Enterococcal bloodstream infections: a 14-year study in a Swiss tertiary hospital. Infect Control Hosp Epidemiol.

[30] Jones RN, Marshall SA, Pfaller MA, Wilke WW, Hollis RJ, Erwin ME, Edmond MB, Wenzel RP (1997). Nosocomial enterococcal blood stream infections in the SCOPE program: antimicrobial resistance, species occurrence, molecular testing results, and laboratory testing accuracy. SCOPE hospital study group. Diagn Microbiol Infect Dis.

[31] Landry SL, Kaiser DL, Wenzel RP (1989). Hospital stay and mortality attributed to nosocomial enterococcal bacteremia: a controlled study. Am J Infect Control.

